# Anxiety, Post-Traumatic Stress Disorder and Social Supports Among Parents of Premature and Full-Term Infants

**DOI:** 10.5812/ircmj.13461

**Published:** 2014-03-05

**Authors:** Maryam Ghorbani, Mahrokh Dolatian, Jamal Shams, Hamid Alavi-Majd

**Affiliations:** 1 Department of Midwifery, Shahid Beheshti University of Medical Sciences, Tehran, IR Iran; 2 Department of Psychiatry, Behavioral Research Center, Shahid Beheshti University of Medical Sciences, Tehran, IR Iran; 3 Department of Biostatistics, School of Paramedical Sciences, Shahid Beheshti University of Medical Sciences, Tehran, IR Iran

**Keywords:** Infant, Premature, Anxiety, Stress Disorders, Post-Traumatic, Social Support

## Abstract

**Background:**

Premature birth is one of the most important unresolved reproductive health problems. Premature birth is often traumatic and a source of distress for parents. Increased parental stress during the first year of their infant's life is a risk factor for later behavioral problems in infants.

**Objectives:**

This study was designed to compare anxiety, post-traumatic stress, and social supports in parents of premature and mature infants.

**Patients and Methods:**

This was a comparative descriptive study conducted at healthcare centers of Qom city, in 2012. In this study, 82 couples (164 parents) divided into two groups including parents who have preterm and term infants. Questionnaires including items such as demographic characteristics, obstetric and post-traumatic stress disorders, Spielberger anxiety and Multidimensional Scale of Perceived Social Support were completed two months after childbirth. Data were analyzed using χ2 test, Fisher’s exact test, Mann-Whitney test, independent t-test, and regression logistic using SPSS18 software.

**Results:**

The levels of anxiety was not significantly different in mothers and fathers in the two groups, but the trait anxiety level of mothers (P < 0.001) and fathers who had preterm infants (P = 0.01) was significantly greater than the parents of full-term infants. Post-traumatic stress disorder was significantly greater in mothers of preterm infants than those of term infants (P = 0.03), but this amount was not significantly different between the two groups of fathers. Mothers' social support did not differ significantly (P = 0.08), however, it was significantly different in fathers (P = 0.01).

**Conclusions:**

Premature infants' parents are more at risk of mental disorders than term infants' parents. This result shows the need of interventions, so these parents can better deal with the problems of premature infants.

## 1. Background

Premature birth is one of the most important unresolved reproductive health problems. Preterm birth is defined as birth after 20 and before 37 weeks of gestational age (1). The prevalence of preterm deliveries in different countries is estimated to be 5 to 13% (1-4). Outcomes of premature infants are related to factors such as status of the baby, parental attitudes, socioeconomic variables, infant’s characteristics, the mother-infant relationship and family environment (5). It seems that the degree to which a mother can overcome the feeling of loss and grief of premature delivery can affect the mother-child relationship (6). Some studies have shown that increased parental stress during the baby's first year is a risk factor for later behavioral problems (7). In addition, pregnancy and childbirth are critical periods in the life of a woman which subsequently induce large changes such as physiological and psychological changes, as well as the social-family roles of women. In addition, the physical changes that occur during pregnancy can boost a woman's emotional instability. Mother’s psychopathological disorders can have psychological effects on child development (8). However, preterm birth is often traumatic and a source of distress for parents. This event is known as an emotional crisis which is often diagnosed with a sense of loss and sorrow. It sometimes lasts for months after the baby discharge from hospital. For many mothers, adjustment with a premature baby is similar to adjustment with a disabled baby (6). When a baby is born normal and healthy, the mother needs to adjust the ideal image she had created of her newborn baby with what she really has. This adjustment is more difficult for mothers of premature infants. Thus, the birth of a premature baby can turn into a stressful emotional experience for most women. These people are mostly at risk of anxiety, even when the infant is clinically healthy (8). Postpartum depression is a common experience in mothers of preterm infants (9). It has been observed that fathers of premature infants had more stress compared to fathers of term infants. During the transition to fatherhood, stressful factors include changes in the role and status of fathers and lack of mothers' attention because of their involvement in the care of premature infants (10).

Social support is an intermediate factor that affects the mental status of people based on World Health Organization framework (11). Perceived social support is individual’s perception of the love and support they receive from family members, friends and acquaintances. Researchers believed that perceived social support is different from received social support (12). There are a lot of studies on the direct and indirect roles (buffer) of perceived social support on reducing stress and improving mental status of individuals. It is believed that social support can directly increase self-esteem, boost resistance against infections, and help to behave in a healthy manner. It can also indirectly cause social adjustment and balance individual’s responses to stressors and reduce stress, which cause to boost the physical and mental health (11, 13-15).

## 2. Objectives

We aimed to compare the anxiety, post-traumatic stress disorder and social support status of parents of premature infants with those parents with term infants.

## 3. Patients and Methods

**Figure 1.**
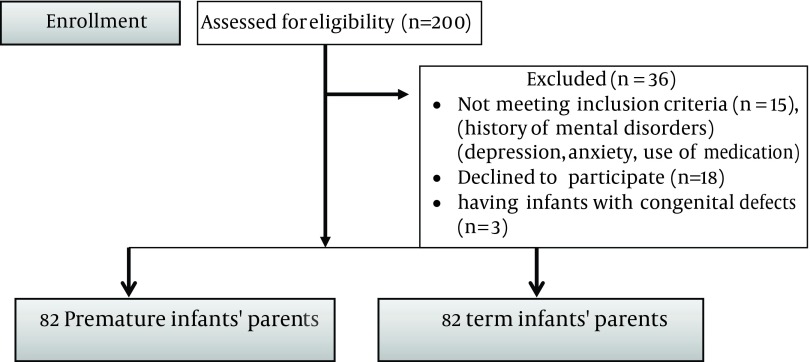
Consort Flow Diagram

This comparative descriptive study was conducted on 164 parents of premature and mature infants who had referred to health centers in Qom city to receive postnatal care from December to March 2012. The inclusion criteria included: no mental illness, no history of any mental disorders, no mental medications usage such as antidepressants, or psychotropic substances, and no neurological or congenital defects in newborns. Based on statistics, each group was comprised of 41 couples (Figure 1), multistage sampling was used in the present study. Firstly, Qom city was divided into four economic classes (the first stage of sampling was cluster sampling). Then all health centers of Qom city were listed and the entire sampling population was distributed based on available data and in proportion to the volume of patients (the second stage of sampling was quota sampling). Then, centers were randomly selected within each region. This study was approved in 20 October 2012 by the Deputy for Research of Shahid Beheshti University of Medical Sciences. To comply with principles of ethics, the following was carried out by the researcher: First, the researcher obtained permission letter from officials at Shahid Beheshti University of Medical Sciences and presented the letter to Qom University of Medical Sciences. During sampling, participation in the study was voluntary, and subjects could decline to continue at any stage of the study. The researcher first introduced herself to participants and explained objectives of the study. Participants were assured of confidentiality of their information. After examination, parents in need of counseling, or treatment were referred to a psychologist or psychiatrist.

The questionnaires for this study include:

Part I:

Questionnaire including father’s information comprised of two parts. The first part includes questions about demographic characteristics, history of psychiatric illness in their first-degree relatives (six questions). The second part comprised of information about the unpleasant events that the father may have experienced during the pregnancy of his spouse (27 questions).

Questionnaire including mother’s information comprised of four parts. The first part includes questions about demographic characteristics, socioeconomic status of the family including housing, family income, and family size (12 items). The second part contained information about the mother's pregnancy such as questions about: previous pregnancies, methods of contraception, prenatal care, the rate of satisfaction of midwife’s care during childbirth, history of diseases during pregnancy, insurance type, delivery type, and hospitalization after delivery (38 questions). The third part included neonatal factors including questions about the baby's gender, type of pregnancy, baby’s disease(s) after birth, infant's history of admission to hospital, parent’s satisfaction with the baby's gender, baby’s gestational age at birth, baby’s weight at birth, baby’s nutrition, problems while caring the newborn, ability to pay baby’s hospitalization costs (15 questions). The fourth part included questions related to unpleasant events in pregnancy (27 questions).

In this study, content validity was used to determine the validity of demographic and obstetric questionnaire. To determine reliability of this questionnaire, test retest analysis was used. The reliability coefficient was calculated as 0.98.

Part II:

Post-traumatic stress disorder symptoms scale:

To diagnose post-traumatic stress disorder (PTSD), the severity of symptoms was evaluated based on DSMIV containing 17 questions with Likert scale. Each question contained a short question and the person’s answers were graded from zero (not at all) to 3 (5 or more times a week). In this scale, the frequency and severity of symptoms have been incorporated because some PTSD symptoms can be evaluated based on their frequencies (such as trauma-related nightmares) and others can be described based on their severity (hyper arousal). Four questions were related to re-experiencing, seven questions were related to avoidance, and six questions were related to motivational responses. In case of one or more re-experiencing related symptoms, three or more symptoms of avoidance, two or more symptoms related to motivational reactions, PTSD was diagnosed (16, 17).

From Iran, Mirzamani et al. studied the validity of this scale using concurrent validity method in 2006 (r = 0.79, P < 0.001). Its test-retest reliability was 0.74 in various studies and 0.88 in Cronbach's alpha method (18).

Part III:

Spielberger Anxiety Questionnaire:

Spielberger Anxiety Questionnaire was performed in order to the study anxiety level. The questionnaire consists of two parts measuring the trait and state anxieties separately. Each section comprised of 20 questions and each question has been scored form 1 to 4. The total score for each individual will be between 20 and 80. Based on this questionnaire, people were classified into three groups of mild (20-40 points), moderate (40-60), and severe anxiety (60-80). This scale has a 0.73 to 0.85 concurrent validity with other anxiety questionnaires (19). Reliability (r = 0.97) and validity of the Persian version of the questionnaire using test re-test was approved in 2007 (20).

Part IV:

Multidimensional scale of perceived social support (MSPSS) was used to measure social support in this research. This scale consisted of 12 items which measured three issues: support perceived by the family members (four items), support perceived by important persons (four items), and support perceived by friends (four items). Based on 7-point Likert scale, all items of this scale ranged from very strongly agree to very strongly disagree. The total score ranged from 12 to 84.

In 2013 Mirabzadeh et al. reported the reliability of this instrument with Cronbach's alpha of 0.89 (21). Sararoudi reported Cronbach's alpha of 0.84 for the scale and 0.90, 0.93 and 0.85, respectively for friends, significant persons and family subscale (22). The samples were selected based on the sample selection criteria by visiting health centers of Qom province (Imam Khomeini, Panbechi, Alzahra and Baqiyatallah Centers). After explaining the research objectives and obtaining the informed written consent from qualified parents of preterm and term babies at the respective centers, demographic and obstetric questionnaires were completed through interviews and also patients’ profiles (to ensure the accuracy of the data). Post-traumatic stress disorder questionnaires, Spielberger anxiety questionnaire, and Multidimensional scale of perceived social support were handed out to the patients. They were asked to fill out the questionnaires immediately and in the presence of the researcher in a quiet environment.

In this study, chi-square test and Fisher's exact test were used to compare the qualitative variables, t-test was used to compare the relationship between quantitative variables, Mann-Whitney test was used for ordinal variables and logistic regression was also used. Data analysis was performed using SPSS version 18. Missing data was replaced by the mean of variables. Confidence coefficient of the study was 0.95.

## 4. Results

The means age of mothers in full-term baby group was 28.22 ± 4.54 and those in preterm infants group was 27.6 ± 6.25, the results showed no significant statistical differences in terms of age between fathers and mothers of term and preterm infants. However, Mann-Whitney test showed that fathers of term infants had higher levels of education compared to preterm infants. This difference was significant (P < 0.001).

In 41.5%, the average monthly salary of fathers in term group was 120 to 240 USD and 46.3 % of preterm group was 240 to 300 USD. As for housing, 37.7 % of the participants owned their place of residence and others had rental housing, governmental housing or lived with their families. The statistical analysis showed no significant difference between the two groups of mothers and fathers in terms of jobs, income and housing type. Most parents in both groups experienced their first childbirth. There were no significant differences between the two groups in terms of labor type, number of childbirths, number of live births, number of sons, number of daughters, abortion history, stillbirth history and infant gender. According to the results, there is a statistically significant difference between infants of two groups in terms of need for hospitalization (P < 0.001) and admission to the neonatal intensive care units (P < 0.001) (Table 1). 

**Table 1.** Demographic and Obstetric Data of Parents of Preterm and Term Infants and Characteristics of Newborns ^a^

Demographic and Pregnancy Information and Characteristics of Newborns	Term	Preterm	P Value
**Mother’s education (High school) **	42 (51.2)	36 (43.9)	> 0.999
**Father’s education (High school)**	24 (29.3)	54 (65.9)	< 0.001
**Mother’s job (housewife)**	69 (84.1)	69 (84.1)	> 0.999
**Father’s job (self-employed)**	57 (69.5)	64 (78)	> 0.999
**Family’s income level**	34 (41.5)	38 (46.3)	< 0.001
**First pregnancy**	37 (45.1)	40 (48.8)	> 0.999
**Number of live births**	39 (47.6)	43 (52.4)	> 0.999
**Abortion history **	15 (81.7)	22 (62.8)	> 0.999
**Stillbirth history**	6 (7.3)	11 (13.4)	> 0.999
**Natural childbirth**	38 (46.3)	47 (57.3)	> 0.999
**Wanted pregnancy from the mother’s point of view**	64 (78)	51 (61.2)	< 0.001
**Wanted pregnancy from the father’s point of view**	64 (78)	47 (57.3)	< 0.001
**Failure of contraceptive methods**	10 (12.2)	23 (28)	< 0.001
**Son (s)**	50 (61)	45 (54.9)	> 0.999
**Need for hospitalization**	9 (11)	48 (58.5)	< 0.001
**Hospitalization at ICU**	3 (3.7)	43 (52.4)	< 0.001
**Afford to pay costs**	80 (97.6)	70 (85.4)	< 0.001
**Son (s)**	50 (61)	45 (54.9)	> 0.999

^a^ Data are presented as No. (%).

No significant relationship was found between disorders such as diabetes, pregnancy hypertension, urinary tract infections, vaginal infections, bleeding or spotting, oligohydramnios, polyhydramnios and other diseases in pregnancy. Overall, there was no significant difference between the two groups in terms of occurrence of unpleasant life events for parents during the last year.

The results showed that in terms of state anxiety, there was no significant difference between the mothers and fathers. However, in the appearance of trait anxiety, the results showed significant differences between the two groups of parents, (P > 0.001). Parents of premature infants significantly suffered from higher trait anxiety than parents of term infants (Table 2). 

**Table 2.** Absolute and Relative Frequency of Trait Anxiety of Mothers and Fathers in Both Groups ^a^

Groups	Term	Preterm	Result Mann-Whitney
**Mothers’ trait anxiety**			P < 0.001
Mild	0	1 (1.2)	
Average	44 (53.7)	72 (87.8)	
Severe	38 (46.3)	9 (11)	
**Father’s trait anxiety**			P < 0.001
Average	61 (74.4)	77 (93.8)	
Severe	21 (25.6)	5 (6.1)	

^a^ Data are presented as No. (%).

The results showed significant statistical differences between the two groups of mothers in terms of PTSD (P = 0.03). The rate of PTSD in mothers of preterm infants is significantly higher than mothers of term infants. The Fisher’s exact test showed no significant difference between the two groups of fathers (Table 3). 

**Table 3.** Absolute and Relative Distribution of PTSD in Parents of Both Groups ^a, b^

Groups	Term	Preterm	χ2
**Mother’s PTSD**			P < 0.001
Yes	1 (1.2)	8 (9.8)	
NA	81 (98.8)	74 (90.2)	
**Father’s PTSD**			P > 0.999
Yes	2 (2.4)	1 (1.2)	
NA	80 (97.6)	81 (98.8)	

^a^ Abbreviation: PTSD, post-traumatic stress disorder; NA, not available.

^b^ Data are presented as No. (%).

The mean perceived social support was 59.49 ± 14.47 and 63.43 ± 14.5 in mothers of term infants, and those of preterm infants, respectively, which shows no significant difference in the perceived social support between two groups of mothers. That for fathers of term babies was 60.6 ± 13.64 and for those of premature infants was 66.43 ± 14.85. Based on the t-test, there were significant differences (P = 0.01) between two groups of fathers in terms of social support. The results of mixed model analysis or GEE analysis are shown in Table 4. 

**Table 4.** GEE Model of Social Support, State and Trait Anxiety, and Post Traumatic Stress Disorder in Parents of Preterm and Term Infants

	Estimate	Standard Error	P Value
**Social Support**			0.006
Term	B = -4.884	1.7786	
Preterm	Reference group		
**Parents**			0.127
Mothers	B = -2.055	1.3469	
Fathers	Reference group		
**State anxiety**			0.817
Term	B= -0.238	1.0295	
Preterm	Reference group		
**Parents**			< 0.001
Mothers	B = -6.409	0.9946	
Fathers	Reference group		
**Trait anxiety**			0.796
Term	B = 0.159	0.6143	
Preterm	Reference group		
**Parents**			0.469
Mothers	B = 0.451	0.6232	
Fathers	Reference group		
**Posttraumatic stress disorder**			0.313
Term	OR = 1.29	0.2366	
Preterm	Reference group		
**Parents**			< 0.001
Mothers	OR = 3.66	0.2237	
Fathers	Reference group		

### 4.1. Social Support

The estimate for the marginal modeling parameter of term variable of -4.88 can be interpreted that mean social support score in the term group had been almost five points less than those in preterm group (P = 0.006). However, in terms of social support scores, no significant difference was found between mothers and fathers (P = 0.127).

### 4.2. State Anxiety

Results revealed no significant difference in the state anxiety between term and preterm groups (P = 0.817). However, the mean level of state anxiety in mothers was 6.41 points less than that in fathers, and this difference was statistically significant (P < 0.001).

### 4.3. Trait Anxiety

Results showed that there was no significant difference between preterm and term groups (P = 0.796). The difference between fathers and mothers in terms of trait anxiety was also not significant (P = 0.469).

### 4.4. Post-traumatic Stress Disorder

Post-traumatic stress disorder was measured using ranking scale, thus, after finding exp (B), the resulting value expressed in the form of OR, could be interpreted as the odds ratio index of 3.66 for variable of parent, which means that the fathers’ chance of post-traumatic stress disorder is almost 3.5 times higher than the mothers. However, no significant difference was found in scores of post-traumatic stress between preterm and term groups (P = 0.313).

## 5. Discussion

Few studies have examined the psychological disorders of parents with preterm babies in early infancy. Most of such studies have mainly focused on the psychological status of mothers while the fathers have been neglected. According to the current study, there was no significant difference between the state anxiety level of mothers in preterm and term groups. Mothers of premature infants experienced significantly higher trait anxiety compared to mothers of term infants. However by using GEE analysis, the state anxiety of mothers was on average 6.41 points lower compared to the fathers, and this difference was statistically significant and the trait anxiety level between fathers and mothers was also similar. In terms of anxiety, the results of this research are not in line with those reported by Gambina et al. in 2011, in terms of stress, anxiety and depression levels in mothers of preterm and term infants (23). Gambina et al. research showed that anxiety of mothers with preterm infants was significantly higher compared to mothers with term infants. The difference in the results might be due to different types of tools used for assessing the anxiety level in these two studies. Gambina et al. found that in mothers of preterm infants stress was significantly higher compared to mothers of term infants. Thus, the results of the present study are in consistent with the results of Gambina et al. According to the current study, mothers of preterm infants had significantly higher PTSD level compared to mothers of term infants. In 2012, Gray et al. conducted a study in Australia to determine the levels of parental stress and mental health in mothers of extremely preterm infants and compared them with mothers of term infants (9). According to their conclusions, the total stress score was not significantly different between the two groups, thus the results of the current study are inconsistent with their report.

The difference between Gray’s research and the present one is that in the current study the questionnaires mentioned above were handed out to parents to evaluate their mental disorders two months after the birth of their baby. However, in Gray et al. research, the data were collected four months after the delivery. They used PSI-SF scale to study mothers’ stress. This is different from the scale used in the present study which is post-traumatic stress disorder scale. Furthermore, Gary et al. only studied the psychological status of the mothers and the fathers’ mental state was ignored. Participants in Gray et al.’s study included mothers who had babies at 24-30 weeks of pregnancy, and their infants were admitted to NICU. In the present study, mothers of infants born between 32-36 weeks entered into the study as preterm group and only 52.4 % of them required hospitalization in intensive care unit.

The findings of the current study are also inconsistent with the those by Yurdakul et al. in 2009 to determine the symptoms of depression, anxiety and mother-infant relationship in neonatal intensive care units (24). According to their report, no differences were observed between the scores of anxiety and type of mother-infant relationship and attachment. Difference between their research and the present study was that in this case-control study, mothers of infants who were admitted to the intensive care unit participated as study group. To pair each mother with her baby in ICU, a mother who had given birth to a healthy term baby considered as control group. Anxiety expression scale was used to study the level of anxiety. Data were collected from the mothers at time of babies’ hospitalization.

Based on the results of the current study, the level of PTSD after childbirth was significantly higher in mothers of preterm infants than mothers of term infants. No difference in PTSD level in fathers of preterm and term babies were detected. Thus, these results are in agreement with those of Pierrehumbert et al. in 2003, which studied the effect of post-traumatic reactions of parents on children’s nutrition and sleep problems, and those of Kersting et al. research that compared the symptoms of PTSD in mothers of preterm and term infants (5, 25). Based on Pierrehumbert et al. study results, parents of premature infants had higher level of PTSD compared with the parents with term babies (5). Furthermore, the rate of prenatal risks increased the risk for PTSD in parents. Based on these results, 67% of mothers showed symptoms of PTSD which was only 6% in the term group.

Severity of parents’ post-traumatic reactions is a strong predictor of the infants’ sleep and behavioral problems. Kersting et al. also found that mothers of preterm infants showed significantly higher rates of PTSD compared to mothers of term group (1-3 days, 14 days, 6 and 14 months after delivery) (25). No reduction was observed in severity of symptoms within 14 months of birth.

Difference between the present study and Kersting study was in data collection and the used tools. They used impact of event scale to measure the signs, and followed mothers up for PTSD symptoms. The difference in the current research with Pierrehumbert research was the time spent on gathering information and the age range of preterm babies. Parents were interviewed about their baby 18 months after delivery, and they were asked to complete prenatal post-traumatic stress disorder questionnaire (PPQ). In their study the premature infant age was between 25-33 weeks; however, in our study the age of preterm infants was 32 to 36 weeks in the present study.

In 2009, Shaw et al. conducted a study to investigate the prevalence of PTSD in parents of premature or sick babies, four months after childbirth to find the relationship between the symptoms of acute stress disorder immediately after birth and PTSD (33.22 % of fathers and 9% of mothers suffered from PTSD). Signs of acute stress disorder were significantly correlated with PTSD and depression. PTSD symptoms appeared in fathers much later than mothers; however, in the fourth month they were at greater risk than mothers and 54.4 % of mothers showed all signs of acute stress disorder while none of the fathers showed such signs (26).

Mean social support score in the term group had been almost five points less than that in preterm group. However, in terms of social support scores, no significant difference was found between mothers and fathers. Social support is an emotional, psychological, informational, and instrumental help provided by others, and directly or indirectly affects the recipient’s behavior. It seems social support for mothers of premature infants compared to those of mature infants is of higher importance because it has been observed that lack of general social support and spouse support is a predictor of maternal distress in mothers with preterm infants (27). Weiss and Chen found a significant relationship between social support provided by friends and family of mothers with preterm infants and their mental health (28).

### 5.1. Final Conclusion

The findings of this study suggest that trait anxiety in mothers and fathers of preterm babies was significantly higher than those in term group. PTSD in mothers of preterm group was significantly higher than mothers of term babies. Regarding the social support of mothers, no significant difference was observed. However, this difference was significant among fathers. Thus, it appears that mothers of preterm infants are at greater risk of mental disorders compared to the fathers. These results show the need for intervention for these parents to better deal with problems of premature infants. The results reveal the existence of mental disorders during this time, and the need of parents’ consultation.

### 5.2. Limitations and Strong Points

When a baby is born prematurely, most of the attention is focused on baby, or at least, mother receives less attention. As is observed in most previous studies in this area, only few studies have focused on the mental status of fathers of these infants, whereas if both parents are considered, it may lead to an improved care of the preterm infant. The main focus of this study was in consideration for fathers’ mental status. Due to the large extent of factors that are associated with mental disorders, all of those could not be covered in this study.
